# COVID-19 Epidemic in the Middle Province of Northern Italy: Impact, Logistics, and Strategy in the First Line Hospital

**DOI:** 10.1017/dmp.2020.51

**Published:** 2020-03-24

**Authors:** Annalisa Gagliano, Pier Giorgio Villani, Francesca M Co’, Anna Manelli, Stefano Paglia, Pietro A. G. Bisagni, Gabriele M Perotti, Enrico Storti, Massimo Lombardo

**Affiliations:** Azienda Socio Sanitaria Territoriale (ASST) Lodi, Department of Surgery, Complex Unit of General and Thoracic Surgery, Lodi, Italy; ASST Lodi, Department of Emergency and Critical Care Unit of Anesthesia and Resuscitation, Lodi, Italy; ASST Lodi, Department of Emergency and Critical Care Unit, Emergency Department, Lodi, Italy; ASST Lodi, Direction and Management, Lodi, Italy

**Keywords:** COVID-19, emergency, public health, strategy

## Abstract

The novel coronavirus (COVID-19) began in China in early December 2019 and rapidly has spread to many countries around the globe, with the number of confirmed cases increasing every day. An epidemic has been recorded since February 20 in a middle province in Northern Italy (Lodi province, in the low Po Valley). The first line hospital had to redesign its logistical and departmental structure to respond to the influx of COVID-19-positive patients who needed hospitalization. Logistical and structural strategies were guided by the crisis unit, managing in 8 days from the beginning of the epidemic to prepare the hospital to be ready to welcome more than 200 COVID-19-positive patients with different ventilatory requirements, keeping clean emergency access lines, and restoring surgical interventions and deferred urgent, routine activity.

## INTRODUCTION

In early December 2019, the first pneumonia cases of unknown origin were identified in Wuhan, the capital city of Hubei province. The pathogen has been identified as a novel enveloped RNA betacoronavirus-2 that currently has been named *severe acute respiratory syndrome coronavirus-2* (SARS-CoV-2), which has a phylogenetic similarity to SARS-CoV.^1^ Patients with the infection have been in the hospital, in family and work environments, and in communities. The World Health Organization has recently declared the novel coronavirus disease 2019 (COVID-19) a public health emergency of international concern.^2,3^ At the end of January 2020, mainland China reported 11 791 confirmed cases of COVID-19 infections that resulted in 259 deaths.^4,5^ Initially, cases were thought to arise from zoonotic transmission; however, recently published literature reveals evidence of human-to-human transmission that increased exponentially by travel, with many cases detected in other parts of the world.^6-8^ This geographic expansion beyond the initial epicenter of Wuhan provides an opportunity to study the natural history of COVID-19 infection.^9^ In relation to the transmission risk of COVID-19,^10^ on January 25, 2020, the Italian Ministry of Health issued the first order with prophylactic measures against COVID-19. After the first one, many ordinances and regulatory circulars defined both prevention measures of behavioral and treatment rules for suspected cases. On February 20, 2020, the first case of SARS-CoV-2 was confirmed in a hospital in Codogno, Italy (a little city of Lodi province in the low Po Valley), hereafter defined as the “red zone” (no entry or leave zone, including 10 cities around Codogno, with quarantine commitment for all citizens). This represented the starting point for extraordinary measures of national and regional management evolving on the basis of the infections registered.

## METHODS

From the first diagnosis, the Emergency Department (ED) of Codogno Hospital was closed to new patients and all routine and elective activity interrupted, “freezing” the hospital at time zero of diagnosis while maintaining the care for patients already hospitalized within the hospital and ensuring their normal care and management. The influx of patients was then diverted to Major Hospital in Lodi, which became the first line hospital. It is necessary to change the hospital organization to manage the eventual epidemic and identify the institutional interfaces. The 2 tracks to follow include the first management of the Territorial Social Health Company (ASST; *Azienda Socio Sanitaria Territoriale*) and the second institutional: region and government. The management of the hospital was entrusted to the crisis unit. Lombardy Region Government transferred to Lodi Major Hospital’s crisis unit complete decision-making power, without the hospital ethics board and master and commander approval. The crisis unit was formed involving hospital staff (general health, nursing), department directors, logistics heads, representatives of critical units in the management of COVID-19 patients, and press coordinator. There was direct collaboration of the prefecture government, law enforcement, civil protection, city, province, and regional governments, as well as indirect collaboration with the National Health Institution, government, and Ministry of Health. The critical points presented at time zero (ie, first diagnosis) arose at various levels:

- *Sanitary*: how many patients in which distribution, with what type of presentation
- *Staff*: who came into contact with others in quarantine who tested positive for SARS-CoV-2, to whom and when to buffer, how many available for the various areas
- *Logistics*: which and how many drugs, which and how many consumer supplies, how many and which ventilation systems, how much oxygen, possibility of rapid re-supplies
- *Structural*: possibility of structural changes within the hospital and any destination use, change, materials, workforce

The main problem immediately encountered was the fluidity and rapidity of requests and the need to change the hospital configuration on the basis of needs. It is almost impossible to redesign physical spaces and restructure management from the beginning. The defined structure was therefore a fixed structure of the management unit (crisis unit) consisting of 2 meetings per day at 10 AM and 4 PM to verify progress and needs and to respond and check the effectiveness of the maneuvers put in place. Major Hospital has 300 beds organized in 9 departments, of which 6 belong to the Health Department, 1 to the Administrative Department, and 2 to the Social Health Department. The Intensive Therapy Department has 7 beds and in non-urgent, routine activity, the ED has near 80 000 access a year with a population of the low Po Valley nearly to 250 000 people. What and how must this be changed?

At the same time, the ED collected data on access and real needs of patients, operating from time zero a carefully and timely collection of epidemiological and health data and initiated a process of change in the management of a triage system.

This model set made it possible to completely reorganize the hospital’s infrastructure in 8 days. We analyzed the changes made in each area and illustrated logistics and needs.

### Emergency Department

**FIGURE 1:**
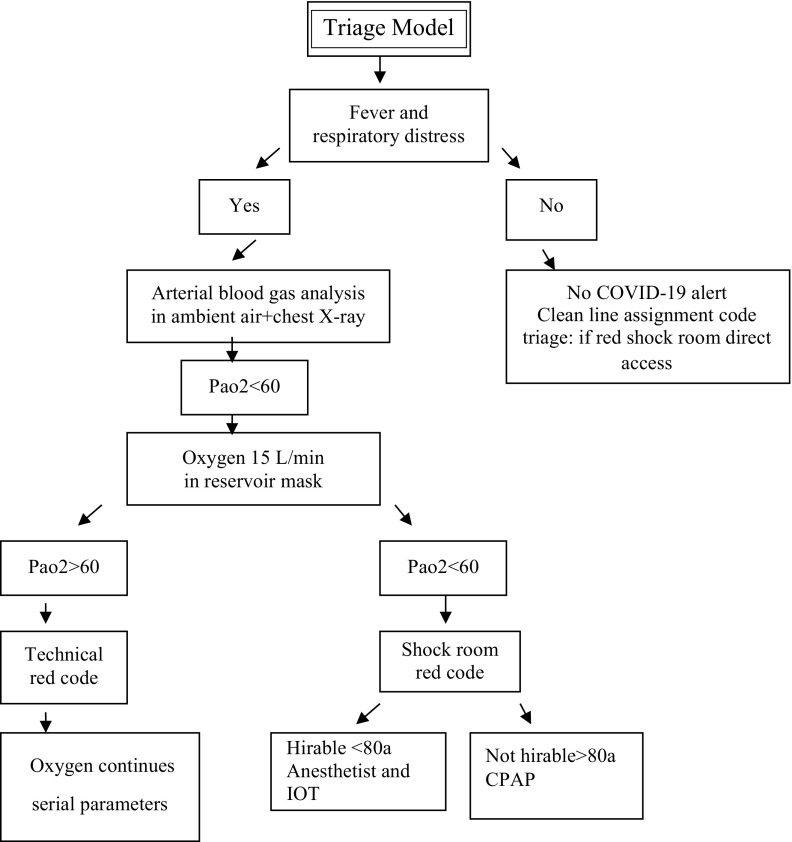
Triage Model in Emergency Department, ASST Lodi.

The director of the ED instituted from the first case a careful data collection of the number and trend of patient access. In the first day, we received patients with a rate of high flux access every 12 hours for a total number of 120. Patients accessed and the trend of treatment were focused on the resolution of acute respiratory failure, but without real knowledge of the presentation of symptoms. The observation in the first days allowed us to identify the common symptoms and signs of the infection, and from this observation the director of the ED designed a model to effectively triage patients and obtain rapid frame rates of respiratory failure and responsiveness of the patients to the oxygen treatment. The director was able to redistribute the ED areas for the different types and needs of patients in scale model and at the same time develop clinical documentation of rapid interpretation to evaluate the improvement or deterioration of the patients (Figure 1), all the while identifying the necessary devices to guarantee the treatment of symptoms.

The evaluation of simplified parameters – temperature, oxygen saturation – was registered in a nominal parameter sheet and attached to the patient’s stretcher and artery blood gas analysis. The schematization and optimization made it possible to identify necessary devices and supplies: oxygen mask, continuous positive airway pressure (CPAP) and ventilation system, stretchers, syringes, personal protective equipment (PPE),^11,12^ and antibiotics. The ED represented the key node for the remodeling of the hospital based on the accesses and the first epidemiological data collected. At the end of the first high influx of patients, we needed 5 intensive care unit (ICU) beds, 20 beds for patients using ventilation support (CPAP), and 40 beds for patients with pneumonia using oxygen support. It’s not possible to wait for the result of the test from a nasopharyngeal swab^13^ (collecting a sample from the back of the nose and throat), because the influx of patients into the ED was at a 12-hour rate and test results came in nearly 72 hours. The ED has been available for non-COVID-19 patients with direct access after triage and dedicated diagnostics, where 2 visiting rooms and a shock room for red codes have been set up.

### Hospital Wards

The health care distribution of the hospital has been completely changed since the third day of the emergency and with fluid progress on the basis of needs. The first measures covered critical areas. But as a first step it was necessary to stop all routine and non-urgent outpatient activity to provide more efficient discharge processing of patients and to free up as many beds as possible in the wards. After the recruitment of beds, it was necessary to change the wards destination and the structural creation of filter zones.^14,15^

- *Intensive care*: from time zero, all patients hospitalized in “sub-intensive” (pulmonary failure or cardiac failure) observation beds were sent to the ordinary ward and such beds were recovered as ICU beds. This allowed increasing the ICU capacity from 7 beds to 17 and predisposing in operating rooms intended for routine minor surgery with available ventilators, lifesaving places for non-viral patients.
- *Wards*: wards and beds have been created for patients with respiratory syndrome. As the first step, an area (at the previous neurology ward, where there were 4 beds monitored and potentially ventilator-equipped) of 18 beds became a critical pneumological area. It was possible to do this in 3 days with structural and logistical changes, creating a filter zone, and defining warehouse and sanitary areas. After these, 2 more 40-bed wards were set up for patients with oxygen needs without the need for intensive or sub-continuous monitoring and again intervening on structure and logistics. At the end of the first week, the hospital completely changed its face. This has also allowed the resumption of deferred urgent activities, including surgical activity.

### Service and Management Structure

- *Radiology*: ED radiology (CT scan, ultrasound, and portable X-ray machine) and conventional X-Ray rooms were considered dirty; the X-ray rooms, the CT scan, and the MRI scan site on the ground floor have been defined as clean and usable for non-COVID-19 patients.
- *Service*: cleaning service has redefined tools to use^16^ and the cycle of service, the kitchen prepared packages with disposable portions for dirty areas.
- *Direction*: permanent crisis unit with scheduled meetings (2 per day at 10 AM and 4 PM)
  – Press office planning and links with prefecture, region, and external law enforcement agencies; connection with Wuhan and with international newspapers; supplies on the basis of the need to renegotiate existing contracts to increase the amount of use of the supplies themselves.
- *Pharmacy*: sorting and controlling use of drugs and PPE; connection with ethics committee for antiretroviral therapy in patients with SARS-CoV-2.

The change was completed, affecting the entire hospital on the eighth day. There was a need for a redistribution of medical and nursing staff for structural changes and to redistribute the staff on duty (not quarantined). Driving permits from the red zone were given to the medical staff of every degree in order to ensure the assistance and the attainment of the workplace.

At the same time, the recruitment of external staff was necessary. There has been voluntary recruitment in the other regional facilities and in the health sector of the Army. Holiday and temporary leave was suspended, and the hospital has been staffed to provide day and night care in all wards. Surgery at all times has guaranteed emergencies with 2 operating rooms activated by day, 1 at night, and guaranteed deferred urgency on a shared operating room between general and specialty surgeries.

## RESULTS

The literature data available at the time of the emergency were few and, above all, stemming from the only experience available on the outbreak from COVID-19. The only country with published data and epidemiological or management studies was represented by the Chinese reality.^9,10,17,18^ However, the health system and the Chinese government represent a very far model from the Italian reality where health is regional and where each ASST has significant autonomy, such as the possibilities available to try to improve and optimize management and logistical choices. Applicable and effective models could result: major-emergency management and military management. Both systems ensure resource optimization in relation to large inflows.^19^ Starting from the concept of advanced medical post and the use of the action plan for the major-emergency, we were able to organize the crisis unit as the hospital’s operations center. At the same time, however, we were not certain about the number of infections or about the real needs of patients. This was the critical point for the initial management. It was therefore necessary to initiate internal analyses that were possible due to the efforts of the ED where epidemiological and health data are recorded up to the first access. After 72 hours, it was therefore possible to have not only the pattern of inflows, but also a realistic prediction of the necessary resources. Every effort was made to have a fluid model inside the hospital. Prospectively, patient management presents a linear management mode for patients who respond to only oxygen therapy: from ED to ward, circular one for the patient in need of ventilator support, from the ED will have to be allocated in 1 of the ventilated areas (depending on the need: ICU or sub-intensive) and then return to the ward. The hospital day by day owing to the data collected and to the great structural and logistical effort was designed as a fluid and circular model. However, the confrontation with the regional government and its health facilities has become decisive and fundamental. In fact, the circular model provides for the need for the involvement of different structures. The first-line hospital, in this case, is represented by a provincial hospital with a capacity limited to 300 beds. The role of the first-line hospital is to ensure framing and need of the patient, but large-scale management of the patients involved must be matched.^20^ This is why the regional network and transfers made it possible not to collapse at the first accesses of the frontline structure. This dynamics model was difficult to realize without reference models and without knowing the number of casualties, and this was not possible to obtain from the beginning; but the presence of all the representations in the management of the crisis has meant that, despite the low sensitivity of prospective data, the model has been applied allowing the management of the first phase of the emergency. In the same way, decentralized management of the first phase in the frontline hospital has allowed other structures, albeit in different ways, to prepare suitable space and line of management for COVID-19 patients.

While in the red zone, the numbers began to grow new oil-spot infections that started to show up in the region, showing the same trend as the Chinese epidemic. At this point, having a management model at our disposal could be the key to better addressing and optimizing the use of resources.

## CONCLUSION

The need for a quick response drove the hospital’s choices for change. Centralizing and managing through the crisis unit has enabled and made change possible. At the end of the first week, the restructuring and the triage and treatment guaranteed by the ED brought the ability of the hospital to manage up to 100 patients in the ED without collapsing and at the same time ensured the management of the patients and the capacity of the hospital according to need. Everything was possible due to the efforts of the medical and nursing staff, who have been redeployed to reinforce critical areas and replace quarantined operators, ensuring maximum assistance.

Coordination of the crisis unit with regional military and government authorities has enabled the centralization of the problem. From the moment that the biohazard emergency represents a certain epidemic, it’s necessary that coordination be increased to a higher level than ASST one and that the “modular” structure can be coordinated at the regional level to ensure increased effectiveness and availability of beds for the health of citizens.

The management on the front line also highlights the need to have a major emergency management plan that is diversified due to the biohazard epidemic, and, in this case, the plan should be regional and agreed in increasing stages of action with a commitment of resources with a structured and centralized model. In this way, both the structures and work units (nurses, doctors, and administrative or health support staff) will be managed as the total account and with progressive commitment. This could also ensure that any infections among the staff are replaced.

Even for logistics, the ability to increase coordination on the basis of total resources would ensure adequate supply as needed, and also cost would be able to preserve the right value. The conclusion of this first phase then creates talking points to improve and optimize the responsiveness of the national health systems.

## Acronyms

Coronavirus disease 2019 (COVID-19)

Continuous positive airway pressure (CPAP)

Intensive care unit (ICU)

Severe acute respiratory syndrome coronavirus 2 (SARS-CoV-2)

Territorial Social Health Company (ASST; *Azienda Socio Sanitaria Territoriale*)
